# Perceptions of agrodiversity and seed-saving practices in the northern Andes of Ecuador

**DOI:** 10.1186/s13002-019-0312-5

**Published:** 2019-07-15

**Authors:** Rommel Montúfar, Michael Ayala

**Affiliations:** 1 Facultad de Ciencias Exactas y Naturales, Pontificia Universidad Católica del Ecuador, Av. 12 de Octubre, 1076 Quito, Ecuador; 2 Facultad de Ciencias Agrícolas, Universidad Central del Ecuador, Jerónimo Leyton s/n y Gatto Sobral. Ciudadela Universitaria, Quito, Ecuador; 3 Latin American & Iberian Institute, University of New Mexico, Albuquerque, NM USA

**Keywords:** Sustainable agriculture, Conventional and non-conventional seeds, Agroecology, Intergenerational agrodiversity loss, Community-based participatory research, Food security and sovereignty

## Abstract

**Background:**

As concerns about agrodiversity loss and its impact on food security increase, interest in seed-saving practices and motivations has risen, especially in regions characterized by ancestral farming. Agroecology practitioners in the northern Andes of Ecuador (*n* = 65) participated in this study to describe (1) the dynamics of intergenerational agrodiversity, (2) perceptions of relevance of the crops they grow, (3) criteria for characterizing the differences between conventional and non-conventional seeds, and (4) their seed-saving practices.

**Methods:**

This exploratory study incorporated a community-based participatory research approach using mixed methods. We conducted (1) a timeline mapping for exploring the dynamics of intergenerational agrodiversity and (2) structured interviews to explore the perception of relevance of crops grown to identify criteria for characterizing conventional and non-conventional seeds and for identifying seed-saving practices. We computed ranks and frequencies from free listing data derived from the interviews to detect the most salient patterns for crop diversity and seed-saving practices. A principal component analysis was performed to illustrate crops distribution within the study area.

**Results and discussion:**

Based on the timeline-mapping tool, we found that participants perceive an intergenerational loss of agrodiversity. Data derived from free listing determined that salient crops differ in each location of the study area, mostly due to geographic (altitude, climate), market factors, and crop management limitations. Responses from open-ended interview questions revealed that farmers discriminate conventional from non-conventional seeds using yield, adaptation to local conditions, pest tolerance, taste, and crop management as criteria. Analysis of free listing data determined that the most salient reported practices related to seed saving were soil fertility management, seed selection, safe seed storage, tilling and rowing, and weeding.

**Conclusions:**

This study contributes to raising awareness of intergenerational agrodiversity loss and replacement with modern crops. We found the relevance of crops and practices is subject to cultural and environmental context, and few agricultural practices are exclusively used for seed saving. Further, farmers clearly discriminate conventional from non-conventional seeds based on advantages and disadvantages, cultural motivation, and produce destination. The community-based participatory approach resulted in positive engagement from participants and promoted commitment from farmers to preserve agrodiversity and support practices at the community level.

## Background

Agrodiversity is a topic of increasing discussion given worldwide concern over climate change, productivity, and food security and sovereignty [1, 2]. The literature defines it as the interactions between agricultural management practices, farmer resources endowments, biophysical resources, and biodiversity within a political context [3–5]. Agrodiversity has gained particular importance in regions with ancestral farming practices due to increases in large-scale farms, agricultural intensification [6], and losses in the appreciation of small-scale farming practices [7]. Many tools and strategies exist to enhance agrodiversity, one of which is the saving of ancestral seeds, mostly based on traditional practices to maintain quality, purity, and germination capacity [8]. A high social–ecological linkage shaped the type of agrodiversity that is most suitable to human consumption patterns and agroecological adaptation of crops [6].

Seed saving is a set of practices oriented towards the conservation and use of genetic material [9] within a political and socioeconomic approach that is sustained on memory, heritage, and ancestral practice [4]. These human practices have been occurring historically for at least 13,000 years, contributing to the evolution of crops according to natural environmental and social dimensions all over the world [10]. Seed saving is more than a careful selection of seeds with the desired traits but also includes seed exchange strategies for disseminating, exchanging, and perpetuating the genetic material. In addition, seed saving has three functions: (1) fostering social bonding among community members and families, (2) maintaining seeds at the household level or in informal markets for preventing loss of genetic material in normal and stress periods, and (3) defining and tracking of genetic diversity patterns [11–14].

Seed saving is an adaptive tool that has sustained ancestral and patrimonial agriculture for millennia around the planet [15]. Activities such as selecting, collecting, storing, and exchanging seeds have helped develop agricultural practices and resource management strategies linked to social relations that helped build both agrarian communities and robust food systems [16, 17]. Case studies from around the world emphasize the community perception of the value of traditional crops as an important component of agrodiversity and the role of particularly complex seed swapping systems that operate among small-scale farmers often in a non-formal regime [14, 18–20]. Long-lasting productive systems historically developed by ancestral agricultural practitioners are now being recovered and reestablished as best practices within a framework called *agroecology*. This discipline contributes to the field with agricultural and natural resource management practices that were adaptatively developed, taking into account natural environmental and social conditions [21].

Agroecology is described as a discipline, practice, and set of actions to achieve social change, challenging conventional agricultural frameworks that have resulted in unsustainable situations for both the environment and humans [22]. The redesign of farming systems using elements of ecology while addressing externalities that had not been considered by conventional agriculture was an early approach to the study of agroecology at the end of the twentieth century [21]. More recently, a framework proposed by Gliessman [23] and later embraced by Miles and colleagues [17] conveys five levels: (1) improving system efficiency by reducing agrochemical use and its ecological and social impact, (ii) substituting unsustainable inputs and practices into agroecosystems, (3) redesigning farming systems with an ecosystem service approach, (4) connecting producers and consumers with a socioecological approach towards the construction of sustainable food systems, and (5) linking equity, participation, and democracy with social justice. In this context, seed saving represents an important contribution to four out of five levels (from 2 to 5) of agroecology.

In the Andes region, the goal of providing sufficient food for the increasing population while preserving natural resources is already a challenge [24]. Pre-Hispanic human societies in the Andean region developed complex production systems with high agrodiversity and seed-saving practices that responded to altitudinal contexts and human dynamics inspired by astronomical and ancestral knowledge [25]. Cultural traditions and community ties remain important in this region today [26]. Presently, these agricultural production systems are facing high climate variability, soil depletion, desertification, pests, and widespread poverty [24]. Additionally, access to efficient markets is a permanent concern of producers, this being an issue that prevents consumers in urban areas from having affordable access to quality food [27, 28]. Despite these challenges for Andean small-scale farmers, there is high potential in recovering ancestral knowledge and practices to tackle climate change, globalized markets, and unsustainable food systems [29].

Being that the Andes region is an important center for genetic diversity of crops [30], research related to strategies that have resulted in high agrodiversity in Latin America is extensive, as is the literature related to on-farm practices for genetic conservation in the region [31–33]. In addition, some researchers have dissected different factors such as: spatial interaction between land use and agrodiversity [34], multifunctionality and political geography of agrodiversity [35], resilience of socioecological dynamics and cultural landscapes [36, 37], social evolution and culture [38–40], and global change [24, 41]. Louette [42] examined the traditional management of seeds and controversies around seed conservation, while Jarvis et al. [43, 44] and Zimmerer [6] assert that modern seeds coexist with traditional ones. Other studies have focused on the impact of farm-level agrodiversity on food systems [2, 45] and the impact of traditional knowledge and practices contributing to sustainability [32]. There is extensive gray literature from development projects and practical experience from rural Latin America, as described by Bellon et al. [46]. Furthermore, the impact of farmers’ practices on genetic diversity, evolution, and strategies for seed conservation have been discussed for emblematic crops like *Zea mays* [33, 47]; nevertheless, seed flows from minor Andean crops have been less covered [48]. Additionally, traditional practices of seed exchange have been reported as social mechanisms that affect the genetic diversity of crops by acting as a selective force in crops such as *Zea mays* and *Oxalis tuberosa* in Mexico and the Andes region, respectively [33, 36, 39, 42, 44, 47–49].

Research outside of Latin America has reported on agrodiversity loss in Africa and Asia, as well as the importance of seed exchanges in trying to maintain the agrodiversity that still exists. For example, van Niekerk and Wynberg [14] depict South African small-scale farmers’ perceptions of the role of social and economic factors in agrodiversity and seed-saving practices, finding that participants report they are experiencing agrodiversity loss. Other studies on Western Africa have portrayed the area’s rich on-farm diversity and its conservation [50]. They also note the importance of cultural bonds and women as major drivers in maintaining agrodiversity in their communities. Farooquee and Maikhuri [19] document perceptions of agrodiversity loss in mountainous regions, specifically in the Himalayas, using surveys with small-scale farmers. However, research within Latin America typically focuses on wild biodiversity, such as in the Amazon Basin and tropical forests [24].

Seed saving is especially important for Ecuador’s current socio-political reality since the publication of the Seeds and Agrobiodiversity Law in 2017 [51]. This regulation establishes new frameworks for agrodiversity use and conservation; seed production, dissemination, and control; and the functioning of production systems and research. The new legislation also provides an official definition for identifying traditional and native seeds and outlines the protocols for certifying conventional seeds. This new reality will certainly affect agricultural and social dynamics in Ecuador regarding seed saving to an extent that is still unpredictable. Consequently, studying those dynamics is of paramount interest in the Ecuadorian Andes to inform both researchers and policy makers about the complexity of these processes. Stromger et al. [33] show a good example of ancestral maize seed systems as a contribution to agrodiversity in the Amazon region; however, there are few similar studies for the Andes region.

In this exploratory study, we aim to contribute to understanding the intersection between agrodiversity and seed-saving practices in Ecuador, as there is limited knowledge in the Andean region around this topic even though it is a major center for crop domestication [52]. We also strive to work with the communities in our study area to engage members and raise awareness of potential concerns, acknowledging the importance of social contexts in maintaining regional agrodiversity. The goals of this study were thus to (1) determine the perceptions of intergenerational ancestral agrodiversity in this region, (2) recognize which crops are most important in community members’ lives and factors affecting this perception, (3) understand participants’ perceptions of conventional and non-conventional seeds, and (4) identify practices related to seed saving and food production and their main drivers. This work presents a case study in the northern Andes of Ecuador, where agroecological practitioners have been recovering ancestral techniques to protect agrodiversity and their best practices related to seed saving. The expectations of this study, based on the literature cited above, are to find evidence of a loss of agrodiversity at a small-scale farmer level and the occurrence of different crops depending on the geographical context. This study also expects to verify the utilization of reliable local knowledge to differentiate conventional from non-conventional seeds and the identification of established seed-saving practices.

## Methods

This exploratory study embraced a community-based participatory research approach, paying respect to the value of local knowledge, assessing the relevance of the topic with the community members, and highlighting that this study is not of them but with them [53]. This research was authorized by the Ethics Committee for Research with Human Beings of the Pontifical Catholic University of Ecuador-CEISH (CEISH-381-2017, October 17, 2017) and was conducted following the code of ethics of the International Society of Ethnobiology (ISE). Additionally, all farmers who decided to participate were interviewed according to mutually agreed conditions. Interviews were done after the participants signed informed consent forms provided in Spanish. Farmers were guaranteed anonymity and were also given the option to discontinue their participation at any time with no consequences.

### Study area

**Fig. 1:**
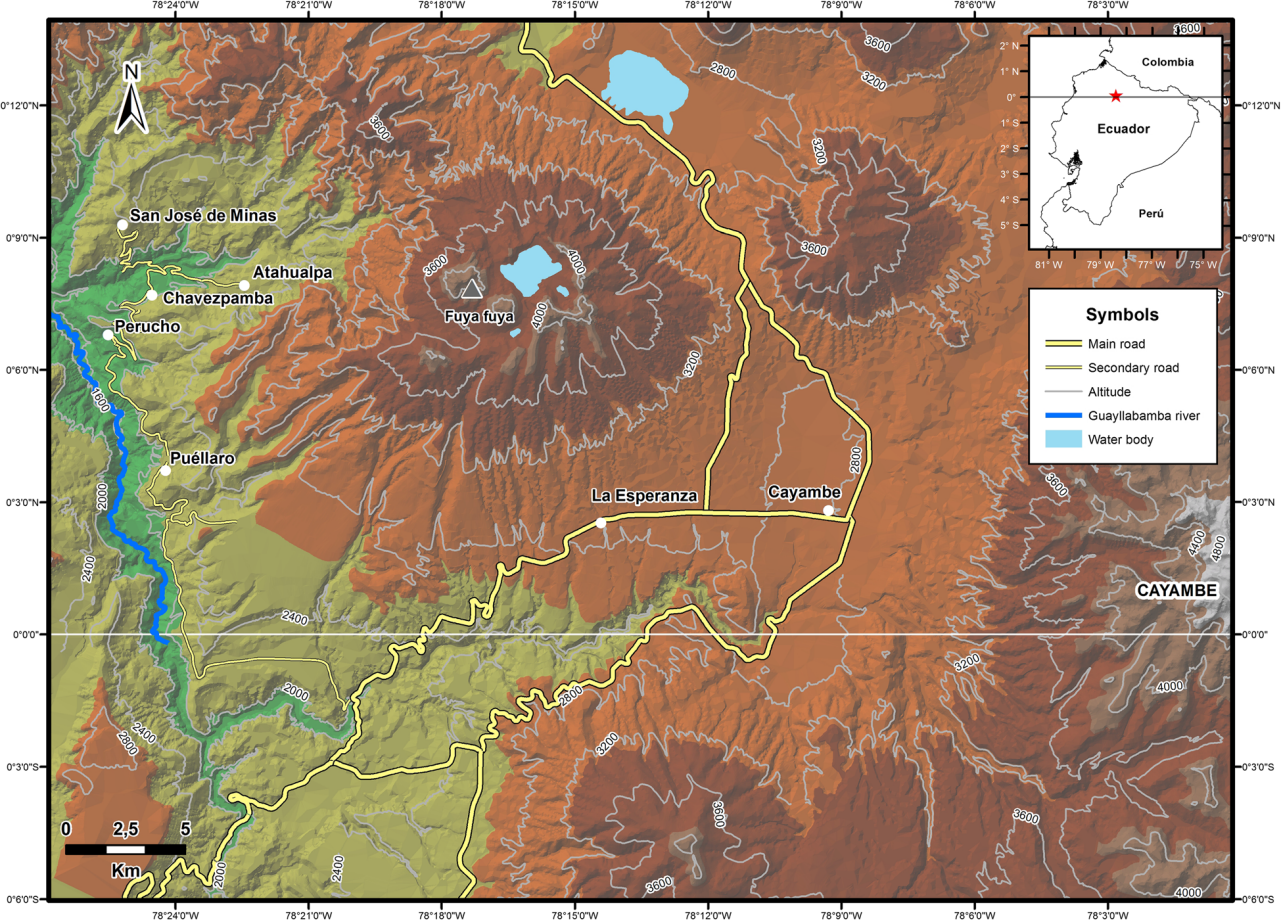
Study area including the localities of Puéllaro, Perucho, Chavezpamba, Atahualpa, and Minas and La Esperanza and Cayambe

This study was carried out in seven localities in the northern Andes of Ecuador. Puéllaro, Perucho, Chavezpamba, Atahualpa, and San José de Minas (hereafter Minas) are in an area historically termed the Peruchana Region, located in the Metropolitan District of Quito [54], and La Esperanza of Tabacundo (hereafter La Esperanza) and Cayambe are outside of Quito in the northern area of the Pichincha province (Fig. 1, Table 1). The localities forming the Peruchana Region are found in a heterogeneous landscape in the high watershed of the Guayllabamba River that includes inter-Andean dry forest and montane forest. This area comprises several altitudinal levels, from 1500 to 2700 masl. The average temperature in Perucho is 17.2 °C, with an annual rainfall of about 767 mm [55]. Due to its topographic heterogeneity, the Peruchana Region has a rich, cultivated biodiversity composed mostly of fruit trees (citrus, avocados, *Annona cherimola*) and root crops (*Arracacia xanthorrhiza*, *Ipomea batatas*) at lower altitudes and cereals and vegetables at higher altitudes. La Esperanza (2889 masl.) and Cayambe (2809 masl.) are located in the inter-Andean valley and are characterized by intense agricultural activity [56]. Agriculture is an important source of income to the household economy in this region, mainly represented by tubers, cereals, and vegetables. The average temperature for Cayambe is 13.5 °C, with an annual rainfall of 873 mm [55]. La Esperanza is only 6 km west of Cayambe, with an average temperature of 13 °C and a rainfall of 832 mm. Both areas have an intensive fresh-cut flower industry that represents a major environmental and social issue [57, 58].

**Table 1:** Demographic characteristics of participants

	Localities					
	Perucho-Puéllaro	Chavezpamba-Atahualpa	Minas	La Esperanza	Cayambe	Total
Altitude (masl)	1951–2088	2217–2280	2400	2889	2809	
Participants	19	13	9	14	10	65
Gender						
Male	12	5	4	6	2	29 (45%)
Female	7	8	5	8	8	36 (55%)
Ethnicity						
Mestizo	19	9	7	7	3	45 (69%)
Indigenous	0	4	2	7	7	20 (31%)
Education						
Elementary	5	10	6	13	8	42 (65%)
High school	11	3	3	0	2	19 (29%)
College	3	0	0	1	0	4 (6%)
Age						
20–39	1	1	2	1	0	5 (8%)
40–49	3	2	1	3	4	13 (20%)
50–59	4	5	2	3	5	19 (29%)
60+	11	5	4	7	1	28 (43%)
Years of farming						
1–9	3	0	0	0	0	3 (5%)
10–29	6	3	3	3	2	17 (26%)
30+	10	10	6	11	8	45 (69%)
Proportion of agriculture in household income						
< 40%	7	3	1	4	3	18 (28%)
> 40% < 60%	4	2	0	2	5	13 (20%)
> 60%	8	8	8	8	2	34 (52%)
Farm size (ha)						
≤ 1	7	12	6	8	8	41 (63%)
> 1 ≤ 3	7	1	2	6	2	18 (28%)
> 3 < 15	5		1			6 (9%)

The ethnicities of the study area’s population are a major feature of this geographical region. Cayambe and La Esperanza have a large indigenous population comprising mostly Kichwa-speaking communities which represent the largest indigenous group, located in the northern Andes of Ecuador. Both localities embrace an ancestral agricultural tradition and are currently recognized as important agricultural centers and consequently harbor high levels of cultivated diversity [59]. The Peruchana Region comprises mostly mestizo communities formed after colonial Spanish occupation. This area does not have a significant proportion of indigenous population [54, 60]. The Peruchana Region is spatially close to the city of Quito (less than 25 km in a straight line), but due to its geographical isolation and poor terrestrial connections, its localities have been enclosed and sometimes totally isolated from urban centers. This situation has historically reduced the community’s access to external inputs, such as modern technology, improved seeds, and synthetic fertilizers. Despite the ethnic and geographical differences, these seven localities are related at a macrolevel because of historical dynamics that favored human flux within this section of the Guayllabamba River’s micro-watershed.

### Participants

Sixty-five small-scale farmers participated in this study. Sampling of the local informants followed two criteria. The first was being an agroecological practitioner, meaning a member of a local association of farmers. Here, we define agroecological practices as those related to improving the efficiency, resilience, multifunctionality, complexity, and integration of the agroecosystem; being equitable and nutritious; and using minimal external inputs [61]. The second criterion was being the head of the family, meaning that they are the farm decision makers and responsible for the family’s well-being (Table 1).

To identify potential participants who matched the first criterion, we built upon our previous community relationships and contacted the leaders of the following farmer associations: Asociación Regional de Soberanía Alimentaria del Territorio Kayambi (RESAK), Asociación de Productores Agroecológicos de La Esperanza, Red de Productoras y Productores Agroecológicos-Cayambe (BIOVIDA), Asociación de Productores de Perucho, and Delegación Norcentral DMQ (corresponding to Puéllaro, Atahualpa, Minas, and Chavezpamba). Leaders were informed about the goals, process, and potential relevance of this project to their communities. The venue, agenda, and purpose of each event were described in an invitation letter that the leaders delivered to community members. Participants were thus the farmers who chose to attend the data collection sessions. Fieldwork data was carried out from October to December 2017.

Seven demographic questions were asked related to gender, ethnic identification, schooling level, age, years of agricultural experience, farm size, and the proportion of solely agriculture income (Table 1). The table illustrates similarities as well as differences among participants. Across all seven localities, the farmers identified themselves as mestizos (69%), but this varied by region. The farmers from Perucho-Puéllaro exclusively identified themselves as mestizos, 50% of the farmers from La Esperanza identified themselves as indigenous, while 70% of the farmers from Cayambe claimed themselves as indigenous. Socio-culturally, farmers from Cayambe and to a lesser extent from La Esperanza speak Kichwa at home, in addition to being fluent in Spanish; the farmers from the other localities speak exclusively Spanish. In our study, most of the farmers (65%) have attended only elementary school (most of them only the first years). This result matches with the average years of schooling (6–9 years) for this region [60]. Most farmers were aged 50 years or older (72%), showing the trend of the aging of rural populations in Ecuador. Household economy mainly depends on agriculture activities (52%); however, 48% considered agriculture as a second source of income. The farming population surveyed was dominated by small-scale farmers with a farm size of ≤ 1 ha (63%). An analysis of poverty level using the Unsatisfied Basic Needs method—which includes indicators of dwelling, overcrowded housing, water and sewerage, education access, and income—reveals that these localities have a high percentage of unsatisfied needs (Minas 77%, Puéllaro 71%, Atahualpa 67%, Chavezpamba 66%, La Esperanza 62%, Cayambe 53%, and Perucho 50%) [60]. This pattern extends to other rural areas of Ecuador and partially explains the limited access to expensive external farming resources (e.g., fertilizers, equipment, technical assistance).

### Data collection

Data collection relied on perceptions and information-gathering with small-scale farmers. This study involved three methods: (1) structured interviews with individuals, (2) a participatory timeline-mapping tool for focus groups, and (3) member validation. Information was collected for both qualitative and quantitative analysis.

#### Structured interviews

Individual interviews in Spanish lasted between 40 and 60 min, took place in community centers, and were carried out by the researchers (who were trained in qualitative research methods). We took detailed notes on participants’ responses for the open-ended questions.

Before conducting the structured interview, we defined key terms for participants. We explained that *seed* refers to all kinds of propagative material (grain, stems, roots, tubers, etc.). We defined *non-conventional seeds* (hereafter NCS) as native and traditional seeds. *Native seeds* were defined as material domesticated by local producers within a community context according to their own knowledge and based on local biodiversity. *Traditional seeds* were defined as domesticated material that is not derived from local biodiversity (exotic) but that has been conserved and adapted to local conditions. *Conventional seeds* (hereafter CS) were defined as those produced and commercialized complying with current Ecuadorian seed law and regulations, regardless of being new acquisitions or native seeds [51].

The structured interview contained six fixed-response and two open-ended questions, which were based on studies with similar research goals [62]:

1. What are the ten most important crops in your gardens? (1 for the most important, 10 for the least important; this question utilized free listing). For each crop reported, how long have you been planting this crop, and where did you get the seeds from? (Choose one: own seeds, seeds from the community, seeds from outside the community.)
2. Do you think that the following criteria determine if a seed is conventional or non-conventional? (1—yes, 2—neutral, 3—no) a, amount of yield; b, the amount of market demand; c, ease of management; d, pest and disease resistance; e, availability; and f, access. Please explain.
3. In what ways are NCS better than CS? (This was an open-ended question.) Please explain.
4. In what ways are CS better than NCS? (This was an open-ended question.) Please explain.
5. What do you do with the produce you grow from NCS? (1—all production, 2—a portion of the production, 3—nothing) a, conventional market; b, agroecological market/baskets; c, bartering; d, industry and exports; e, gift; and f, self-consumption. Please explain.
6. What do you do with the produce you grow from CS? (1—all production, 2—a portion of the production, 3—nothing) a, conventional market; b, agroecological market/baskets; c, bartering; d, industry and exports; e, gift; and f, self-consumption. Please explain.
7. What ten practices do you consider the most important so that you can have good seeds? (1 for the most important, 10 for the less important; this question utilized free listing).
8. To what extent does each of the following factors (climate, available technology, market, personal knowledge, community organization, and local/national regulations) prevent or encourage the implementation of the practices you mentioned for saving good seeds mentioned in question 7. (Choose one: encourage, prevent, or both.) Please explain.

#### Participatory timeline mapping

The participatory timeline-mapping exercise was used to explore perceptions of agrodiversity through time. This tool, adapted from Kolar et al. [63] to create visual historic narratives, assisted the facilitation of a structured interview with focus groups, bringing contextual depth to the narrative. Timeline mapping is a visual tool for representing facts that happen over time, providing a historical representation of a phenomenon. This mapping allows participants to enhance rapport, interaction, engagement, and agreements. Maps produced in the process work both as a memory aid and visual guide for further analysis [63]. In the present study, varieties reported by farmers were analyzed as taxonomic units.

In the case of this study, participants historically represented the crops used at three different moments. This allowed for the active participation of farmers who produced narratives and discussion about the evolution of agrodiversity in the area. Specifically, three timeline-mapping data collection exercises were held, involving participants from all locations: one focus group with 40 farmers representing the Peruchana Region, the second with 18 farmers from Cayambe, and the third with 12 farmers from La Esperanza. Farmers were invited to picture themselves within the context of their farms in the past, the present, and the future. Participants aged 40 years and older were encouraged to actively contribute to this exercise because of their insights into older crops. For clarity, we asked participants to decide on which name they preferred for each plant to reduce the chance of misrepresentation or name confusion. We asked them to answer the following questions:

1. What are the crops you remember were planted by your grandparents in their gardens? (past)
2. From the crops you already mentioned, which ones are you currently planting in your gardens? And what else do you plant? (present)
3. From the crops planted by your grandparents, which ones would you like your grandchildren to plant in the future? And what other crops do you hope they plant? (future)

#### Member validation

Member validation, or respondent validation, added credibility to our data collection and analysis [64]. We invited all participants to a focus group, with 25 attending a 2-hour session held as the final data collection activity. We showed the participants the preliminary results and asked them to look for any information that was incorrect or missing or names of plants that were misrepresented. This procedure contributed to the spirit of community-based participatory research.

### Qualitative and quantitative analysis

Both qualitative and quantitative analysis methods were carried out for this study. Prior to analysis, criteria for classification of vegetables and fruits were established, related to their uses and dietary purposes. Classifications can be different from region to region according to criteria of seasonal availability, degree of ripeness and cultural culinary uses [65]. For the purpose of this study, we considered vegetables as a large range of edible plants that are (1) mostly used fresh, (2) mainly served raw, and (3) generally short-term crops. The definition of vegetable in this study excludes staple foods such as *Solanum tuberosum*. The vegetable category includes broccoli, cabbage, onion, cilantro, celery, carrot, beets, spinach, lettuce, bell pepper, radish, cucumber, chard, cauliflower, romanesco, and parsley. For this study, we grouped lentils, fava beans, chickpeas, and peas under the denomination of legumes. Although beans of all kinds are technically included in the legumes group, for this study, we considered beans as a separate category because of their large variability and occurrence in the study area. The classifications of the crops between native and exotic follow the descriptions provided by de la Torre et al. [52].

#### Structured interviews

The interview questions were analyzed by question type.

##### Question 1

For the first question about the ten most important plants in their gardens, we followed the analysis implemented by Puri [66], transforming a free list into a matrix of crops (rows) and respondents (columns), recording the answers (1 to 10) of all interviewees. The average rank (the sum of all ranks within a row/number of respondents that listed that item) and frequency (number of times it occurs across respondents/total number of respondents) were computed for each crop recorded. A plot using average rank (*y*-axis) and frequency (*x*-axis) of the crops recorded was created to visualize the more salient seed species. Only crops with frequencies larger than 5% were included in the graph. For this question, farmers reported plants as species, which were then analyzed as taxonomic units.

To detect local patterns among the most ecologically differentiated localities, we computed average ranks and frequencies for each one of these data sets and again graphed frequency and average rank as above. The communities chosen for this analysis were Perucho and Puéllaro, communities which are close together and share a similar dry and mountainous environment, vs. La Esperanza, which is more humid and has a flatter landscape.

Additionally, we conducted a principal component analysis (PCA) to use as a tool to visualize general patterns. The compiled data were structured into a binary matrix (presence or absence) including only crops with frequencies greater than 5% (descriptors). We entered farmers’ data (*n* = 65) into the analysis as observations and crops as descriptors (*n* = 40). A bi-plot describing the multidimensional patterns was carried out plotting the first two eigenvectors. We carried out the PCA using XLSTAT software (Microsoft version 2016). Information related to time of use of these crops and their origin was presented using averages, standard deviation, and coefficient variation values.

##### Question 7

The analysis for question 7 followed the same procedure as for question 1 (free listing) regarding plotting the average rank (*y*-axis) and frequencies (*x*-axis) of the 21 practices that lead to having good seeds for frequencies larger than 10%.

##### Questions 2–6 and 8

The results of questions 2–6 and 8 were depicted using histograms.

#### Timeline mapping

The localities were grouped into three areas due to similarities in geographical context and identity: Peruchana Region (Perucho, Puéllaro, Chavezpamba, Atahualpa, and Minas), Cayambe, and La Esperanza. We tabulated a list of crops for each time period (past, present, and future) and location to visualize the intergenerational variations in terms of crop composition (see the “Appendix” section). Those values were depicted in a bar graph for each of the three groups.

## Results and discussion

We worked with 65 participants, mainly people older than 60 years, representing the local rural reality in the territory. This number may appear to be low, but it represents an important proportion of agroecological practitioners in our study area. Working with older participants was an advantage because they are experienced farmers and are more likely to have been exposed to traditional crops and to have witnessed the dynamics of agrodiversity on their fields. In contrast to Mier y Terán et al. [67], who assert that agroecological knowledge and practices are taken up by younger generations, in our study area, we observed that traditional seed-saving and agrodiversity knowledge are mainly preserved by older practitioners [68].

### Perceptions of intergenerational ancestral agrodiversity

**Fig. 2:**
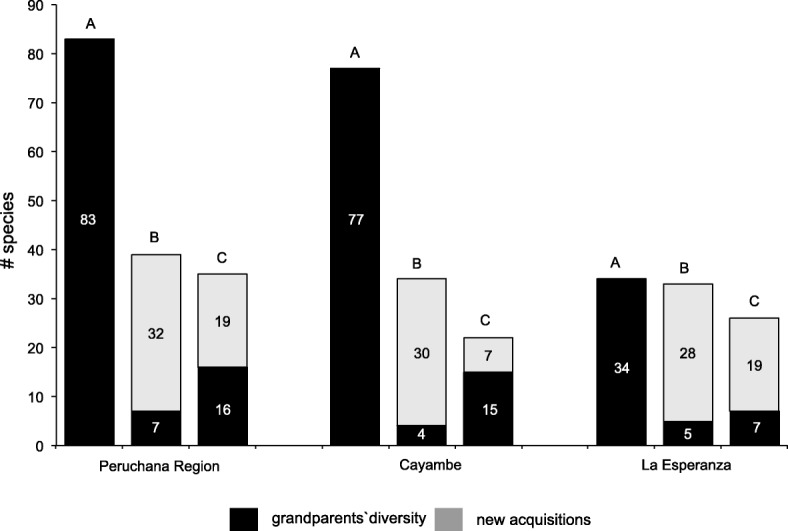
Intergenerational agrodiversity of crops among the past (grandparents, A), the present (us, B) and the hopes for the future (grandchildren, C) in three areas of Ecuador’s northern Andes. Black depicts grandparents’ agrodiversity, and gray depicts newer acquisitions

The first goal of this study was to describe perceptions of intergenerational ancestral agrodiversity in the northern Andes, focusing on potential changes over time. The timeline-mapping tool revealed that participants perceived that there had been vast intergenerational ancestral agrodiversity loss concurrent with replacement of these crops with modern ones (Fig. 2). A list of the 239 taxonomic units and their scientific names are included in the “Appendix” section.

Figure 2 illustrates the differences in the proportion between what participants remember their grandparents planting vs. what they plant. The diversity reported for the grandparent’s farms in the Peruchana Region was for a total of 83 crops, around 71% of them native plants. The most represented crops were *Ipomoea batatas* (*n* = 15 varieties), *Zea mays* (*n* = 9), *Phaseolus vulgaris* (*n* = 6), *Solanum tuberosum* (*n* = 4), and *Arracacia xanthorrhiza* (*n* = 4). Currently, participants plant only seven out of the 83 crops from their grandparents, which represents a perceived loss of 92% of agrodiversity compared to their grandparents. Interview responses indicated that they could not plant these crops because the seeds are no longer available, they do not know how to farm these crops, or they are unsure of how to cook with this produce; they also noted that some of the older varieties have been replaced by newer hybrid crops. They hoped their grandchildren would grow 16 out of the original 83, focusing on varieties of *S*. *tuberosum*, legumes, *Saccharum officinarum*, *Psidium guajava*, *Musa* sp., and *Chenopodium quinoa* that are no longer grown but that they remember from their childhood. We assume that conventional market pressure, new food consumption tendencies, and the implementation of modern agricultural technology makes farmers expect their grandchildren to grow newly acquired and exotic seeds in addition to the traditional ones [40, 69].

A similar pattern was reported for Cayambe, where the grandparents’ farms were reported to have 77 crops (native crops comprising 48 which represent 62% of all crops reported). The most represented crops were *S*. *tuberosum* (*n* = 5) and *Ageratina dendroides* (*n* = 3). Currently, farmers plant only four of these crops in their gardens, which represents a 95% loss. They hoped that their grandchildren would be able to grow 15 out of the 77 crops their grandparents grew, focusing on varieties of *Lupinus mutabilis*, legumes, *Myrica pubescens*, *Z*. *mays*, *Tropaeolum tuberosum*, *Ullucus tuberosus*, *Ch*. *quinoa*, and herbs. It is important to highlight that *A*. *dendroides* is generally undervalued in the northern Andes; however, in the study area, it was consistently reported as a crop. For La Esperanza, we found less agrodiversity compared to the previous locations (native crops comprising 20 which represent 59% of all crops reported). The farmers there reported 34 different crops at their grandparents’ farms. The most reported crops were *S*. *tuberosum* (*n* = 4), *Z*. *mays*, *P*. *vulgaris*, and *Hordeum vulgare* (*n* = 3 for each). Currently, participants plant five out of these 34, which represents an 85% loss. They hoped their grandchildren would be able to plant only two more crops than they currently grow (legumes and *Oxalis tuberosa*).

The perception of loss in ancestral agrodiversity over time is evident. This pattern matches a large-scale spatial analysis of diversity present in farmers’ fields described by the FAO [68] and similar studies that have focused on areas where local inter- and intra-specific ancestral agrodiversity has been diminished, especially in areas of Ecuador where cash crops have been established with modern agricultural systems [69]. In contrast, some studies have reported that traditional crops are not only being maintained by small-scale farmers but also being increased [6, 44]. This augmentation is not the case in our study area or for our spatial analysis, where the evident reduction of historical diversity and the replacement of local diversity for a few modern crops have been occurring. It is also palpable that these participants hope their descendants will regain some of this loss. The fact that these participants only hope that their grandchildren would grow a small portion of the plants grown by their grandparents, however, raises questions regarding these farmers’ realities. This study was unable to ascertain to what extent a lack of knowledge or appreciation of the old crops, pragmatic concerns over available space, etc., might be affecting their hopes for their grandchildren’s gardens and thus ongoing local ancestral agrodiversity.

### Crop importance in farmers’ lives

**Fig. 3:**
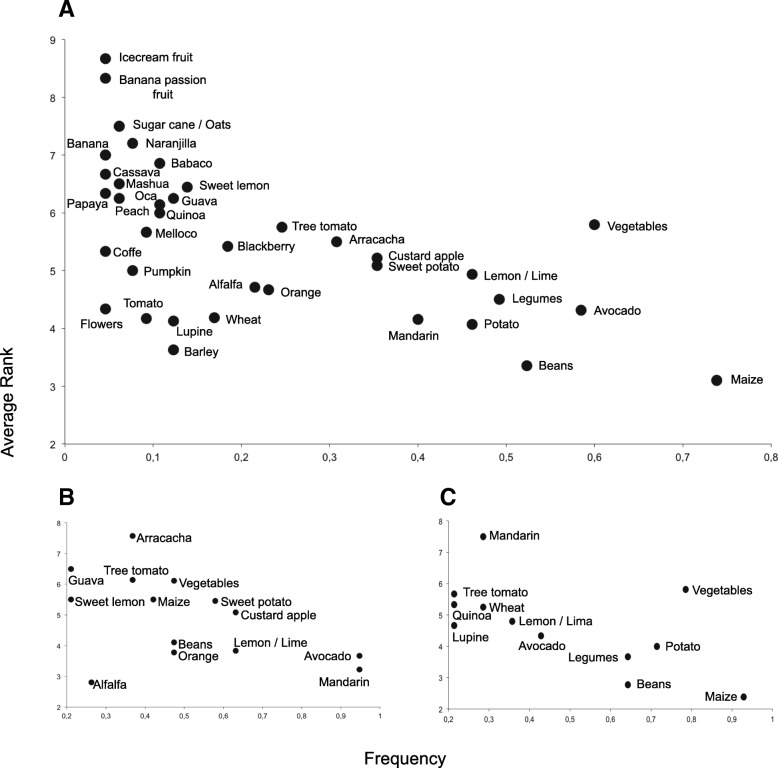
The most salient crops. Average rank (1 to 10) versus frequency. **a** All samples (*n* = 65); only crops with a frequency > 5% were included. **b** Farmers from Perucho and Puéllaro (*n* = 19); only crops with a frequency > 5% were included. **c** Farmers from La Esperanza (*n* = 14); only crops with a frequency > 5% were included in the figure. The right bottom represents the most salient crops and the left upper the lesser salient crops

Providing an overview of which crops are most important in farmers’ lives and factors affecting this perception were the second goal of this study. In response to interview question 1, related to the ten most important crop seeds managed in their gardens, the 65 farmers reported 61 crops. Figure 3a summarizes the major patterns detected in the study area. The items placed on the lower right-hand corner represent the crops mentioned more frequently and listed in a higher rank in the free list; in contrast, the crops located on the top left represent the crops mentioned infrequently and listed in a lower rank in the free list.

Figure 3a reveals that *Z*. *mays*, *Persea americana*, vegetables, *P*. *vulgaris*, and other legumes (*Cicer arietinum*, *Vicia faba*, *Lens culinaris*, and *Pisum sativum*) were, in general, the most salient crops. However, the most important crops differed according to each location. For instance, Fig. 3b shows the most important crops managed in the areas of Perucho and Puéllaro (*n* = 19): *P*. *americana*, *Annona cherimola*, *I*. *batatas*, *Citrus limon* (exotic), and *Citrus reticulata* (exotic). Due to its warmer climate, particularly in the lowest areas (1750 masl.), these locations have developed an agricultural tradition mainly based on fruit trees such as citrus and *P*. *americana*. Farmers from the Peruchana Region have developed their particular propagation material and management protocols in a traditional fashion.

On the other hand, the most important crops reported for La Esperanza (*n* = 14) were *Z*. *mays*, vegetables (mostly exotic), *S*. *tuberosum*, *P*. *vulgaris*, and other legumes (Fig. 3c). This result raises concerns since native and highly nutritious Andean crops such as *C*. *quinoa*, *Amaranthus quitensis*, *L*. *mutabilis*, and Andean roots and tubers like *A*. *xanthorrhiza*, *T*. *tuberosum*, and *O*. *tuberosa* were not stated by farmers as their most important crops. Based on narratives obtained on question 8, we suggest that the reasons why those native crops are not salient in their farms are (1) for roots and tubers, lesser market demand, and (2) for *L*. *mutabilis*, *C*. *quinoa*, and *A*. *quitensis*, post-harvesting limitations. Our findings are supported by reports from Kichwa indigenous villages in the Chimborazo province [40], where farmers claim that nutritious Andean crops have a secondary presence on small-scale farms.

**Fig. 4:**
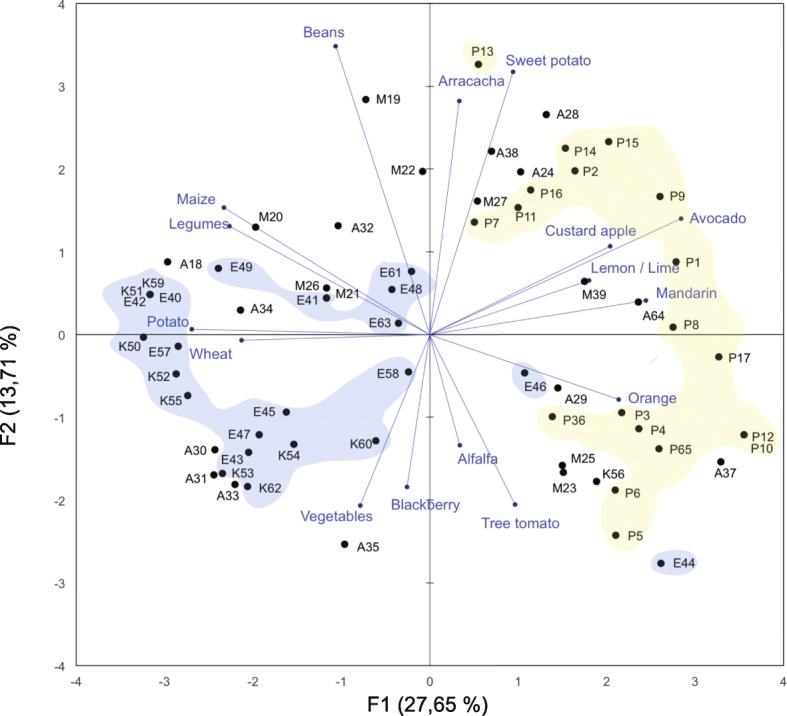
Principal components analysis (PCA) for 65 farmers included in this study based on the most relevant crops for their farms. The farmers with the prefix P are from Perucho and Puéllaro, K from Cayambe, E from La Esperanza, A from Atahualpa and Chavezpamba, and M from Minas

A PCA bi-plot (axis 1 = 27.65%; axis 2 = 13.71%) was created to describe the total variation and the main crops that characterize all the study area (*n* = 65 farmers; Fig. 4). Axis 1 in Fig. 4 is mostly related to an altitudinal gradient where the farmers from the lowest locations, Perucho and Puéllaro, are placed mostly on the right side of the biplot, whereas the farmers from La Esperanza and Cayambe appear on the left side, corresponding with the higher altitude. The remaining farmers from Chavezpamba, Atahualpa, and Minas are scattered in the PCA bi-plot. Farmers from Perucho and Puéllaro are characterized by *C*. *reticulata*, *P*. *americana*, *I*. *batatas*, *A*. *xanthorrhiza*, *C*. *limon*, and *C*. *cherimola*. Meanwhile, *Z*. *mays*, vegetables, *S*. *tuberosum*, *Rubus glaucus*, *Triticum vulgare*, and legumes characterize the majority of farmers from La Esperanza and Cayambe. This result apparently responds to an association of the composition of crops with altitude, and consequently, with climate.

Table 2 shows the length of time that crops have been planted: for example, *T*. *vulgare*, *Z*. *mays*, *P*. *vulgaris*, *S*. *tuberosum*, *I*. *batatas*, and legumes have been planted for more than 30 years. These are NCS crops with over 30 years of planting tradition. Results are coherent with the history of this region where *Z*. *mays*, *S*. *tuberosum*, legumes, and *I*. *batatas* are native and traditional crops, which are typically disseminated among farmers. *Triticum vulgare* was historically cultivated and then lost in the region due to massive wheat donations from the USA starting in 1954 [70], but today, public initiatives are trying to recover the local wheat diversity. The results shown in Table 2 should be cautiously interpreted since the average number of years planted for most of the crops has a high standard deviation.

**Table 2:** Years planting the same genetic material and its origin. This list only includes crops with a frequency higher than 10 out of the 65 small-scale farmers

Crops	*n* ^a^	Average number of years planted		Origin of seeds *n* ^b^		
		($\overline{x}$ ± SD)	CV (%)	Own	Community	Out of community
*Zea mays*	48	34.60 ± 19.88	57.46	29	8	9
Vegetables	39	13.03 ± 16.29	125.07	9	3	27
*Persea americana*	38	15.26 ± 14.37	94.17	6	7	24
*Phaseolus vulgaris*	34	31.26 ± 21.52	68.84	25	3	5
Legumes	32	29.50 ± 20.01	67.82	23	2	5
*Solanum tuberosum*	30	29.80 ± 22.10	74.15	17	5	7
*Citrus limon*	30	13.30 ± 12.92	97.12	8	6	16
*Citrus reticulata*	26	19.15 ± 18.81	98.23	7	9	9
*Ipomoea batatas*	23	29.52 ± 24.11	81.68	17	3	0
*Annona cherimola*	23	20.65 ± 18.62	90.18	8	5	9
*Arracacia xanthorrhiza*	20	20.60 ± 24.60	119.40	9	6	4
*Solanum betacea*	16	12.00 ± 13.91	115.91	4	2	9
*Citrus sinensis*	15	19.20 ± 17.44	90.84	6	2	7
*Medicago sativa*	14	10.14 ± 12.54	123.62	1	0	13
*Rubus glaucus*	12	7.58 ± 7.91	104.36	3	1	6
*Triticum vulgare*	11	40.27 ± 15.61	38.76	10	0	1

*CV* coefficient of variation, *n*^a^ number of respondents for years using same genetic material, *n*^b^ number of respondents for seed origin

The most salient crops reported, which are also NCS (*Z*. *mays*, *P*. *vulgaris*, *S*. *tuberosum*, *I*. *batatas*, and other legumes) come from seeds produced at participants’ own farms or from their relatives. These findings are consistent with the social dynamics of Andean culture, which are based on strong family and community bonds [26, 49]. For instance, the seed exchange for *Lupinus mutabilis* in the Ecuadorean Andes is strongly linked to a legacy and tradition in which local farmers prefer their own seeds or seeds from close neighbors [39]. A similar pattern was reported by maize farmers in Jalisco, where only 15% of the land planted with maize could be attributed to exotic introductions [42]. Further, between 75 and 100% of the seeds used by farmers in Aguaytia (in the Peruvian Andes) was exchanged within the community, which seems to be a regular pattern [31]. However, the seed exchange ratio could be much larger. In contrast, propagation material for other crops like vegetables, *P*. *americana*, *C*. *limon*, and *M*. *sativa* are obtained outside the community because those are mainly cash crops, relatively new in the area (less than 15 years) and mostly, but not necessarily, CS. Seeds exchanged within the community showed no evident pattern. Narratives produced during the interviews lead us to suggest that this behavior responds to a blurry frontier between community and family assets in the study area. This result reveals that the seed system is fairly local, as has been previously described for Amazonian [44] and Andean communities [48].

**Fig. 5:**
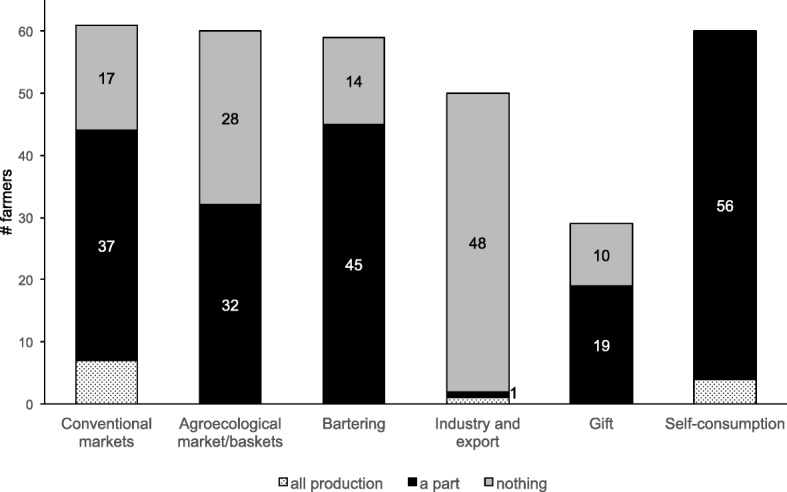
The destination of the produce from CS and NCS

We also investigated the destination of the produce from both CS and NCS from the following six categories: conventional markets, agroecological markets, bartering, industry and exportation, gift-giving, and self-consumption (Fig. 5).

Of the 61 farmers who plant NCS, only 40 reported that they also plant CS. Four farmers indicated they were unaware of the source of their seeds. Proportionally, the destination between both groups was similar, with the main difference being in the practice of bartering and the second being in gift-giving; more farmers barter with and give gifts with their NCS than CS. Bartering involves an exchange of goods (seeds) of an agreed value; this exchange usually occurs in seed-swapping events or farmers’ markets. With gift-giving, however, the nature of the gift does not involve an exchange of goods with an agreed value. One local farmer said, “I give away plants or seeds to someone who is going to actually plant them,” and another said, “We only give away the very best of produce.” According to these narratives from participants, we can conclude that gift-giving enhances the maintenance of both high-quality food and propagation materials. Both bartering and gift-giving are practices that help disseminate seeds and perpetuate agrodiversity [31, 44], and these practices also promote food security in areas with short food supply chains. For instance, Zimmerer [36] reported that more than 95% of potatoes and maize seeds in western South America come from this informal exchange. Similar patterns were reported for rice seeds in Nepal in 1999–2000 [31]. Although all of the farmers claimed to be agroecological for this study, more than 50% of their produce goes to conventional markets, mostly because not all the seven localities included in this study have agroecological farmers’ market or some other alternative ways of marketing produce. Industry and export as a destination for produce are underrepresented, mostly because agroecological production in these localities is oriented towards food autonomy of the communities. Farmers rarely use their entire production for a single goal. Narratives from respondents endorse the social role of both bartering and giving seeds and produce as a strategy for increasing and maintaining agrodiversity in their farms and for strengthening community and family bonding [13, 14, 26].

### Participant perceptions of NCS vs. CS

**Fig. 6:**
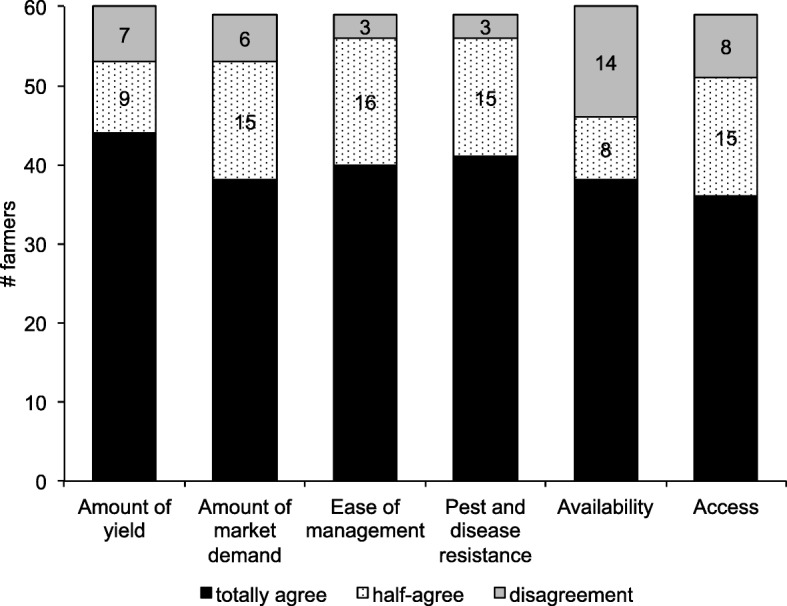
Criteria for discriminating between CS and NCS based on 60 farmers’ response

The third goal of this study was to describe participants’ perceptions of conventional and non-conventional seeds. For the purpose of this study, NCS refer to native and traditional seeds and CS to seeds with a conventional management regardless of those materials’ origin, either native or new acquisitions, as stated in the methods section. Figure 6 shows responses to the question if participants think that certain criteria determine if a seed is conventional or non-conventional, with fixed responses being yes, neutral, or no. The criteria were (1) amount of yield, (2) amount of market demand, (3) ease of management, (4) pest and disease resistance, (5) availability, and (6) access.

Most of the farmers agreed that the six proposed criteria synthesize the major differences between NCS and CS. The first potentially discriminating factor was amount of yield. Research in other regions has shown that there are no clear conclusions regarding productivity between CS and NCS. Epule and Bryant [71] and Heilig and Kelly [72] argued that older bean cultivars (*P*. *vulgaris*) had a lower performance compared to modern commercial cultivars regardless of the production system, although some genotypes showed a better performance under organic production systems. On the other hand, Northwest Washington heirloom dry beans showed a double average yield compared with standard commercial varieties in the Columbia Basin [73]. Productivity can vary across a large number of factors depending on the nature of the crops and species as well as the different environmental conditions, the crop’s conservation status [74], and human behavioral factors. The aforementioned human factors that affect productivity, including more attention paid by agroecological growers to small-scale gardens compared to commercial plots, could also explain differences in productivity and are related to farm size [71, 73].

Another potentially discriminating factor is market demand. The narratives reveal that the farmers think that NCS crops have a better market opportunity as long as there is a farmers’ market and/or a food movement that focuses on organic, local, or traditional dishes in the vicinity, such as in La Esperanza and Cayambe. This social–cultural linkage between consumption and traditional seed use has been previously depicted in Bolivia [6] and in northern Ecuador [25]. The development of local markets represents the key to recovering local traditional food, increasing farm-level income, fostering social dynamics, and protecting agrodiversity, which has been consistently described in literature from the USA, Brazil, and Peru [33, 75, 76]. The market setting plays a paramount role in promoting seed flows and hence boosting agrodiversity [36, 48]. The role of these types of agroecological initiatives and social factors in the ancestral agrodiversity of a region is an area that merits further research.

Regarding the discriminating factor of ease of management, the farmers reported that it is easier to manage NCS in the field than CS—mainly because these seeds are more adapted to local climate, soil, and environmental conditions and because NCS have an increased tolerance and resistance to pest and diseases (criterion 4). This long-term in situ selection has been largely reported in the literature [77].

Participant comments regarding the fifth (availability) and sixth (access) criteria reveal that NCS are perceived as easier to buy and find than CS. We can hypothesize that economic and cultural constraints [14, 26] can drive the farmers’ behavior when obtaining the seeds. Participants stated that the availability of economic resources restricts the capacity of low-income farmers to buy CS at the seed store; consequently, CS are less accessible for the farmers. In this context, farmers prefer to get NCS, which are more accessible via familiar and community networks and have a lower price or are free [78]. However, we cannot attribute the farmers’ decisions only to economic factors. Culture (with its values, memories, legacies, and principles) also plays a key role in their decisions, as has already been explored in the Andean communities of Ecuador [26]. Perceptions of the differences between these types of seeds gain importance given that few studies are available on this topic in the Andes region in general.

#### Ways in which NCS are better than CS

**Fig. 7:**
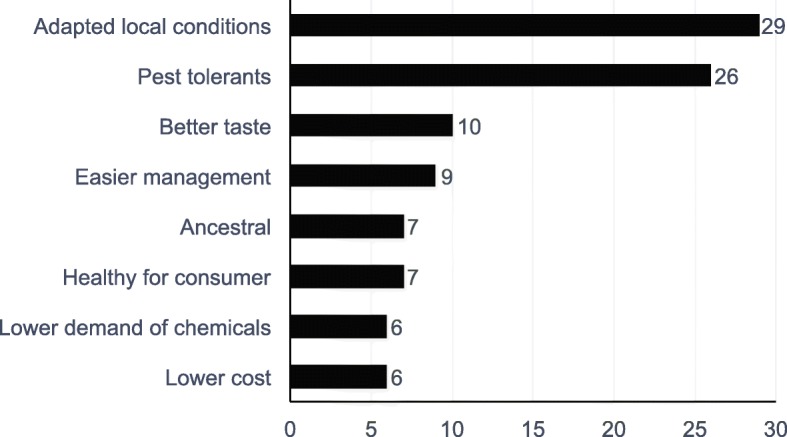
Ways in which NCS are better than CS. A total number of 21 attributes were identified. Only the criteria with a frequency ≥ 5 are represented in this figure

The 65 farmers provided 21 ways in which NCS are better than CS (Fig. 7). Participants noted two major ways in which NCS are different to CS: positive interaction with environmental conditions (adapted to local conditions, greater pest tolerance, ease of management, lower demand of chemicals) and sociocultural-economic factors (better taste, ancestral heritage, healthy for consumer, lower cost). NCS’ adaptation to local environmental conditions has been largely described in the literature [21]. Even further, the literature describes adaptation of crops to local socioeconomic and cultural contexts. For instance, in South Africa, where mostly genetically modified crops (GMOs) are grown, CS are best suited for large-scale monocrops, mostly oriented to the market, whereas NCS are best suited for small farmers with less access to mechanization and external farming resources [14]. Chorol et al. [79] describe the better adaptation of NCS to socio-environmental contexts illustrated with a study in the Himalayas where NCS, specifically heirloom root vegetables, had a longer shelf life and contributed to food security during the winter months.

The other preference for NCS over CS by farmers corresponds to sociocultural-economic factors. Better taste of NCS produce, which can be categorized as a sensory quality, was mentioned. A perception of superior taste contributes to the acceptability of NCS as food at the community level, and sensory qualities can include other elements such as color and shape [79] This perception is aligned with one of the factors of food security definitions which is socio-cultural acceptability. Participants produced narratives where they stated that traditional and ritual meals taste better with ingredients from NCS. However, there are few well-controlled studies on sensory qualities that have made a valid comparison between NCS and CS. Regarding NCS being healthier for consumers, participants based this opinion on nutritional and safety factors. For instance, heirloom fig trees (*Ficus carica*) from southern Arizona home gardens showed less vitamin A, but potassium values were significantly higher [80]. Ancestral heritage was another reason given for preferring NCS, since community members link these seeds with their Andean heritage and identity, as described by the Kichwa communities near our study area [26]. Narratives showed a feeling of ownership of NCS, which contributes to the construction of identity. Finally, NCS adaptation to environmental conditions results in easier management, and consequently those crops require fewer pesticides, which makes them safer to consumers, as stated by participants.

#### Ways in which CS are better than NCS

When farmers were asked about the ways in which conventional seeds are better, the main reasons were economic: (1) increased production (*n* = 18), (2) better quality (*n* = 10), (3) faster growing cycle (*n* = 9), (4) easier to market (*n* = 8), and (5) less economic risk (*n* = 6) (17 other reasons were presented with lower frequencies). Surprisingly, local farmers are aware that CS facilitate a better, more productive, and market-oriented production yet still choose NCS. One of the multiple explanations for this apparent contradiction is related to health. Health concerns are a particular issue in this area. Specifically, Cayambe and La Esperanza have been exposed to the harmful effects of industrial agriculture (flower farms), the effects of which have been reflected in the increased incidence of chronic and fatal diseases [56, 81].

### Practices for seed saving and food production

**Fig. 8:**
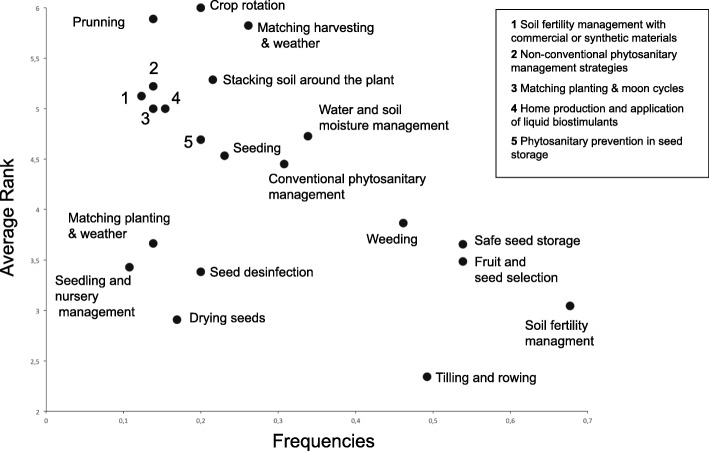
The most important seed-saving practices in the study area

The fourth aim of the study was to examine practices related to seed saving and food production in this region. Using the free listing tool, 65 respondents produced narratives that were coded into 41 seed-saving practices. Figure 8 depicts only practices with a frequency higher than 0.1. The *y*-axis represents average rank and the *x*-axis represents frequency. Practices located in the lower right corner were the most salient for both frequency and average rank. These practices are (1) soil fertility management, (2) fruit and seed selection, (3) safe seed storage, (4) tilling and rowing, and (5) weeding. The most common practices are reported in all localities despite their different environmental conditions. Nevertheless, when separately analyzing Perucho and Puéllaro (*n* = 19), concern for water and moisture management was reported as the second most important priority after soil fertility, which is a typical concern for regions with a dry climate. Additionally, for these two locations, two distinct activities were mentioned: selection of best individuals for rootstocks and selection of plants for grafting buds. This individual analysis demonstrates that there are commonalities applicable to all locations, but there are also particularities based on local environmental conditions. Narratives portray practices for seed saving being the same ones implemented for food production, with only six practices directly related to seed-saving processes: (1) seed storage, (2) fruit and seed selection, (3) seedlings for vegetable production, (4) disinfection, (5) drying seeds, and (6) seedlings and nursery management for fruit tree production. From the first set of processes mentioned above, only “seed storage” directly matches the results of other seed-saving studies in Latin America [32]. In addition, the same authors depict practices such as “native seed conservation” and “family seed banks,” which are related to the “seed storage” practices that have already been reported in our study. Meanwhile “seed adaptation to environment” described by Parraguez-Vergara et al. [32] coincides with some of the categories reported in our study, including general management practices like “matching harvesting and weather” and others. Narratives from participants reported that seed exchange practices such as produce swaps, bartering, and gift-giving include plant parts and botanical seeds that are utilized both as seeds and food. These practices directly contribute to seed perpetuation in a non-explicit fashion and enhance food security at the local scale. Direct seed exchange has been reported in Latin America as being common in local and regional contexts [32] as well as inherent to the local culture [49].

#### Drivers of seed-saving practices

**Fig. 9:**
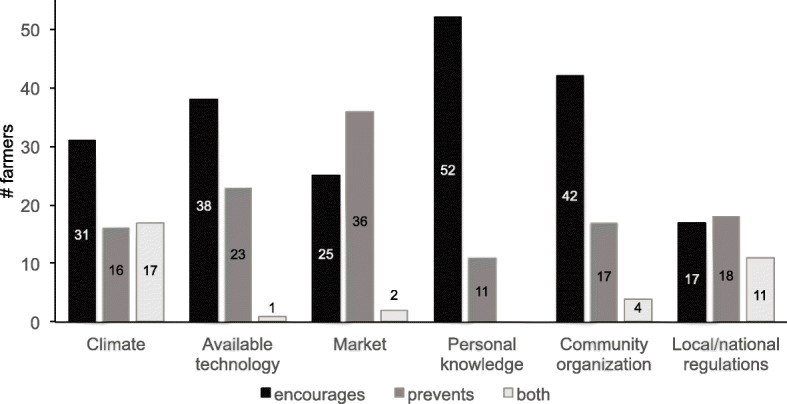
The extent to which the following factors (climate, available technology, market, personal knowledge, community organization, and local/national regulations) prevent or encourage the implementation of the seed-saving practices mentioned in question 7

The farmers from the study area agreed that climate, available technology, personal knowledge, and community organization encourage the implementation and execution of high-quality seed-saving practices. The market was the only factor mentioned as discouraging the implementation of these practices (Fig. 9). Local and national regulations were not mentioned as having either a positive or negative influence.

The communities showed high self-confidence about their current knowledge and how they apply that knowledge to their farms, which was related to the long tradition of local farmers in the study area. In the case of participants from La Esperanza and Cayambe, which comprise mostly indigenous communities with spiritual bonds to their land, their ancestral knowledge about their land was more evident in their narratives than in the other localities. Gondard and Lopez [15] describe landscape interventions in the Cayambe territory meant to adapt to climate and topography that can be traced to pre-Incan occupation. Conversely, the farmers in the Peruchana Region, who identify themselves as mestizos, have more recently acquired knowledge, evidenced, for example, by the irrigation systems derived from the Spanish (acequia systems). The Peruchana Region’s geographical isolation from the Quito Valley due to the Guayllabamba River canyon favored the development of local capacities and natural resource management approaches adapted to its specific geographical conditions, including the irrigation systems brought by the Spaniards, in areas lower than 2000 masl (Vinicio Ayala-Delegación Norcentral Municipio del Distrito Metropolitano de Quito, personal communication).

Community organization was recognized as an important factor that contributes to the implementation of high-quality seed-saving practices. Social structures like farmers’ markets, agricultural cooperatives, women organizations, and credit unions influence farmers’ daily activities and decision-making processes. Most farmers affirm that the current available technology, including equipment and facilities, are enough to carry out their productive activities. Finally, climate was identified as a positive factor for applying seed-saving practices; however, an important proportion of respondents assert that climate may both prevent and promote that implementation. Narratives from participants portray the growing concern about the negative impacts of climate change on agriculture. Even farmers from higher altitude areas, above 2000 masl where there has not been the need for irrigation in the past, expressed their increasing requirement for water. This and other issues related to climate change, which demand adaptation strategies in the high Andes for small-scale farmers, have been largely discussed in the literature [24]. Regarding local and national regulations, they do not show a particular pattern of influence in the application of practices for seed saving. However, in some locations like La Esperanza, farmers recognized that regulations from their rural organizations have important effects on their day-to-day activities. One regulatory system that is becoming more widely implemented—the Participatory Guarantee System (PGS), required to guarantee farmers’ compliance with agroecological principles—will likely influence the application of high-quality practices in this region [82].

As the market was the only factor they identified as having a negative impact on their practices, it can be determined that the current market prices of agroecological produce are not sufficiently high to stimulate the production of agroecological crops, except in areas with recognized local farmers’ markets. This finding is consistent with other research that has shown that consumers in areas that lack an identifiable market for this type of produce are unaware of the value of agroecological food [83]. La Esperanza and Cayambe do have their own farmers’ markets, which promote the trade of agroecological produce in an alternative and short supply chain as described by Marsden et al. [84] in a general context. Nevertheless, an element that more fully contributes to this marketing strategy is the local PGS they developed. The aim of this system is to build mutual trust, encouraging engagement between producers and consumers [82]. The absence of a reliable short marketing circuit is a real concern especially for the Peruchana zone; this type of market issue has been described as the bottleneck for agroecological initiative development [83].

## Conclusion

This exploratory study is one of the first of its kind to document agroecological small-scale farmers’ perceptions related to agrodiversity practices in the northern Andes of Ecuador. Whereas many other studies describe realities in countries in Africa or Asia, this research fills a need in the literature as it focuses on Latin America. Specifically, this study documents the community’s perceptions of intergenerational ancestral agrodiversity loss in this region; provides an overview of which crops are most important in their lives and the factors affecting these perceptions, such as the geographical context; describes the knowledge used by local farmers to differentiate conventional from non-conventional seeds; and presents established practices undertaken in this region related to seed saving and food production. These findings confirm the original expectations of this study. Local small-scale farmers in this region perceive that they are experiencing a loss of intergenerational agrodiversity due to many factors including a loss of access to ancestral seeds as well as current market demands that encourage the production of conventional crops. In regions studied with agroecological farmers’ markets, conventional concerns are somewhat mitigated, leading to increased hope for upward trends in ancestral agrodiversity in the future. Undertaking a community-based participatory research study, we were able to raise the awareness of local farmers in these Andean communities about their changing agrodiversity and food options, engage them in the research process, and contribute to an increase in their commitment to preserve agrodiversity and supporting practices.

Although this study was focused on perceptions of local small-scale agroecological practitioners, perceptions of conventional farmers would enrich the discussion in future studies. We are also aware that the study area is not totally representative of the whole Andean region, therefore, we recommend replicating similar studies in other geographical areas. Future research should incorporate a morphological and genetic characterization of local agrodiversity, including longitudinal studies that can document trends over time. Ethno-botanically based research is needed, which will provide an understanding of how communities use these crops in their lives. Given the realities of Ecuadorian laws related to ancestral seeds and crops, future research can build on our findings and further contribute to deeper understandings of distinctions between CS vs. NCS.

## Data Availability

The datasets used and/or analyzed during the current study are available from the corresponding author on reasonable request.

## Appendix

**Table 3:** List of crops (239 taxonomic units) reported in the time line mapping

Taxonomic unit	English name	Scientific name	Family	Localities		
				Peruchana Region	Cayambe	La Esperanza
Acelga	Chard	*Beta vulgaris* L.	Amaranthaceae	x		
Achira		*Canna edulis* Ker Gawl.	Cannaceae		x	
Achogcha	Achojcha	*Cyclanthera pedata* (L.) Schrad.	Cucurbitaceae	x		
Aguacate	Avocado	*Persea americana* Mill.	Lauraceae	x	x	x
Aguacate nacional	Avocado	*Persea americana* Mill.	Lauraceae	x		
Aguacate tipo fuerte	Avocado	*Persea americana* Mill.	Lauraceae	x		
Ají chawa	Chili	*Capsicum annuum* L.	Solanaceae		x	
Ají rocoto amarillo	Chili	*Capsicum pubescens* L.	Solanaceae		x	
Alcachofa	Artichoke	*Cynara scolymus* L.	Asteraceae	x	x	
Alfalfa	Alfalfa	*Medicago sativa* L.	Fabaceae	x		
Aliso		*Alnus acuminata* Kunth	Betulaceae		x	
Alverjón		*Pisum sativum* L.	Fabaceae	x		x
Amaranto blanco	Amaranth	*Amaranthus quitensis* Kunth	Amaranthaceae		x	
Anku-yuyu		*Muehlenbeckia tamnifolia* (Kunth) Meisn.	Polygonaceae		x	
Apio	Celery	*Apium graveolens* L.	Apiaceae	x	x	x
Arquitecta		*Aetheolaena patens* (Kunth) B. Nord.	Asteraceae		x	
Arrayán		*Myrcianthes* sp.	Myrtaceae		x	x
Arveja	Pea	*Pisum sativum* L.	Fabaceae	x	x	x
Arveja enana	Pea	*Pisum sativum* L.	Fabaceae			x
Arveja paramera pequeña	Pea	*Pisum sativum* L.	Fabaceae	x		
Ashkumikuna /cebadilla		*Avena fatua* L.	Poaceae		x	
Hatum-Sara		*Phytolacca bogotensis* Kunth	Phytolaccaceae		x	
Babaco	Babaco	*Vasconcellea × heilbornii* (V.M. Badillo) V.M. Badillo	Caricaceae	x		
Badea		*Passiflora quadrangularis* L.	Passifloraceae	x		
Borraja	Boraje	*Borago officinalis* L.	Boraginaceae		x	
Brócoli	Broccoli	*Brassica oleracea* L.	Brassicaceae	x		
Café	Coffee	*Coffea arabica* L.	Rubiaceae	x		
Camote baños	Sweet potato	*Ipomoea batatas* (L.) Lam.	Convolvulaceae	x		
Camote cargamento de dulce	Sweet potato	*Ipomoea batatas* (L.) Lam.	Convolvulaceae	x		
Camote cargamento de sal	Sweet potato	*Ipomoea batatas* (L.) Lam.	Convolvulaceae	x		
Camote chivo	Sweet potato	*Ipomoea batatas* (L.) Lam.	Convolvulaceae	x		
Camote chufata	Sweet potato	*Ipomoea batatas* (L.) Lam.	Convolvulaceae	x		
Camote de dulce	Sweet potato	*Ipomoea batatas* (L.) Lam.	Convolvulaceae	x		x
Camote de sal	Sweet potato	*Ipomoea batatas* (L.) Lam.	Convolvulaceae	x		
Camote morado	Sweet potato	*Ipomoea batatas* (L.) Lam.	Convolvulaceae	x		
Camote negro	Sweet potato	*Ipomoea batatas* (L.) Lam.	Convolvulaceae	x		
Camote pata de gallina	Sweet potato	*Ipomoea batatas* (L.) Lam.	Convolvulaceae	x		
Camote peseta	Sweet potato	*Ipomoea batatas* (L.) Lam.	Convolvulaceae	x		
Camote puka	Sweet potato	*Ipomoea batatas* (L.) Lam.	Convolvulaceae	x		
Camote quitumbe	Sweet potato	*Ipomoea batatas* (L.) Lam.	Convolvulaceae	x		
Camote urpe	Sweet potato	*Ipomoea batatas* (L.) Lam.	Convolvulaceae	x		
Camote yumbo	Sweet potato	*Ipomoea batatas* (L.) Lam.	Convolvulaceae	x		
Camote zapallo	Sweet potato	*Ipomoea batatas* (L.) Lam.	Convolvulaceae	x		
Camotes	Sweet potato	*Ipomoea batatas* (L.) Lam.	Convolvulaceae		x	
Caña de azúcar	Sugar cane	*Saccharum officinarum* L.	Poaceae	x	x	
Capulí		*Prunus capuli* Cav.	Rosaceae		x	x
Cartuchos		*Zantedeschia aethiopica* (L.) Spreng.	Araceae			x
Cebada	Barley	*Hordeum vulgare* L.	Poaceae	x	x	x
Cebada chimba	Barley	*Hordeum vulgare* L.	Poaceae			x
Cebada grande	Barley	*Hordeum vulgare* L.	Poaceae			x
Cebada llucha	Barley	*Hordeum vulgare* L.	Poaceae		x	x
Cebolla amarilla	Onion	*Allium cepa* L.	Amaryllidaceae		x	
Cebolla blanca larga	Onion	*Allium cepa* L.	Amaryllidaceae	x		
Cebolla colorada	Onion	*Allium cepa* L.	Amaryllidaceae		x	x
Cebolla paiteña	Onion	*Allium cepa* L.	Amaryllidaceae	x		
Cebolla paiteña híbrida	Onion	*Allium* sp.	Amaryllidaceae	x		
Cedrón	Cedron-Verbene	*Aloysia gratissima* (Gillies & Hook.) Tronc.	Verbenaceae		x	x
Centeno	Rye	*Secale cereale* L.	Poaceae	x		
Chamburo		*Vasconcellea* sp.	Caricaceae	x	x	
Chawarpenco	Maguey	*Agave americana* L.	Asparagaceae		x	
Chia		*Salvia hispánica* L.	Lamiaceae		x	
Chilca brillante		*Ageratina dendroides* (Spreng.)R.M.King &H.Rob.	Asteraceae		x	
Chilca hembra		*Ageratina dendroides* (Spreng.)R.M.King &H.Rob.	Asteraceae		x	
Chilca macho		*Ageratina dendroides* (Spreng.)R.M.King &H.Rob.	Asteraceae		x	
Chirimoya	Custard apple	*Annona cherimola* Mill.	Annonaceae	x	x	x
Chihualcán		*Vasconcellea* sp.	Caricaceae	x	x	
Chocho	Lupine	*Lupinus mutabilis* Sweet	Fabaceae	x	x	x
Chocho blanco con negro	Lupine	*Lupinus mutabilis* Sweet	Fabaceae			x
Ciruelo		*non identified*		x		
Claudia		*Prunus salicina* Lindl.	Rosaceae	x		
Clavel	Carnation	*Dianthus* sp.	Caryophyllaceae		x	
Col común de mata	Cabbage	*Brassica oleracea* L.	Brassicaceae	x		
Coliflor		*Brassica oleracea* L.	Brassicaceae	x		
Culantro	Cilantro	*Coriandrum sativum* L.	Apiaceae		x	x
Congona		*Peperomia galioides* Kunth	Piperaceae		x	
Durazno	Peach	*Prunus pérsica* (L.) Batsch	Rosaceae	x	x	
Escancel morado		*Alternanthera* sp.	Amanranthaceae		x	
Espinaca	Spinach	*Spinacia oleracea* L.	Amanranthaceae	x		x
Frailejón		*Espeletia* sp.	Asteraceae		x	
Fréjol	Bean	*Phaseolus vulgaris* L.	Fabaceae	x		x
Fréjol bayo	Bean	*Phaseolus vulgaris* L.	Fabaceae			x
Fréjol bayo azul	Bean	*Phaseolus vulgaris* L.	Fabaceae	x		
Fréjol bayo canario	Bean	*Phaseolus vulgaris* L.	Fabaceae	x		x
Fréjol campeón	Bean	*Phaseolus vulgaris* L.	Fabaceae	x		
Fréjol familiar guiador	Bean	*Phaseolus vulgaris* L.	Fabaceae	x		
Fréjol guiador	Bean	*Phaseolus vulgaris* L.	Fabaceae	x		
Fréjol magolita	Bean	*Phaseolus vulgaris* L.	Fabaceae	x		
Fréjol ocre	Bean	*Phaseolus vulgaris* L.	Fabaceae			x
Fréjol Popayán	Bean	*Phaseolus vulgaris* L.	Fabaceae	x		
Fréjol rojo guiador	Bean	*Phaseolus vulgaris* L.	Fabaceae	x		
Fréjol shayandero	Bean	*Phaseolus vulgaris* L.	Fabaceae			x
Fréjol Toa	Bean	*Phaseolus vulgaris* L.	Fabaceae	x		
Garbanzos locales	Chickpea	*Cicer arietinum* L.	Fabaceae			x
Granada	Pomegranate	*Punica granatum* L.	Lythraceae	x		
Granadilla	Passion fruit	*Passiflora edulis* Sims	Passifloraceae	x		x
Guaba	Icecream fruit	*Inga edulis* Mart.	Fabaceae		x	x
Gualicón		*Macleania* sp.	Ericaceae	x		
Guayaba blanca	Guava	*Psidium guajava* L.	Myrtaceae	x		x
Guayaba rosada	Guava	*Psidium guajava* L.	Myrtaceae	x	x	
Guayabilla	Guava	*Psidium guajava* L.	Myrtaceae	x		
Guineo guaytarilla	Banana	*Musa* x *paradisiaca* L.	Musaceae	x		
Guineo limeño	Banana	*Musa* x *paradisiaca* L	Musaceae	x		
Guineo rosa	Red banana	*Musa* x *paradisiaca* L	Musaceae	x		
Haba blanca	Fava bean	*Vicia faba* L.	Fabaceae			x
Haba chaucha	Fava bean	*Vicia faba* L.	Fabaceae			x
Habas	Fava bean	*Vicia faba* L.	Fabaceae	x	x	
Habas grandes	Fava bean	*Vicia faba* L.	Fabaceae	x		
Habas místicas	Fava bean	*Vicia faba* L.	Fabaceae			x
Habas sangre de Cristo	Fava bean	*Vicia faba* L.	Fabaceae			x
Habichuela		*Vicia faba* L.	Fabaceae	x		
Habilla		*Vicia faba* L.	Fabaceae	x	x	x
Hierba buena	Hierbabuena	*Mentha spicata* L.	Lamiaceae			x
Hierba maggi		*Apium* L.	Apiaceae		x	
Hierba mora		*Solanum nigrum* L.	Solanaceae		x	
Higo	Fig	*Ficus carica* L.	Moraceae	x		
Ibilán		*Monnina obtusifolia* Kunth	Polygalaceae	x		
Jícama	Jicama	*Smallanthus sonchifolius* (Poepp.) H.Rob.	Asteraceae		x	x
Yurak panka		*Gynoxys* sp.	Asteraceae		x	
Juyanguilla		Not identified			x	
Kiwi	Kiwi	*Actinidia deliciosa* (A. Chev.) C.F. Liang & A.R. Ferguson	Actinidiaceae		x	
Laurel		*Myrica pubescens* Humb. & Bonpl. Ex Willd.	Myricaceae		x	
Lenteja buena	Lentil	*Lens culinaris* Medik.	Fabaceae			x
Lenteja musga	Lentil	*Lens culinaris* Medik.	Fabaceae			x
Lenteja zuca	Lentil	*Lens culinaris* Medik.	Fabaceae		x	x
Lima	Sweet lemon	*Citrus limon* (L.) Burm.f.	Rutaceae	x		
Limón	Lemon	*Citrus limon* (L.) Burm.f.	Rutaceae	x	x	x
Linaza	Linseed	*Linum usitatissimum* L.	Linaceae		x	
Maíz amarillo	Maize	*Zea mays* L.	Poaceae	x	x	x
Maíz blanco para choclo o mote	Maize	*Zea mays* L.	Poaceae	x		
Maíz canguil blanco	Maize	*Zea mays* L.	Poaceae	x	x	
Maíz canguil negro	Maize	*Zea mays* L.	Poaceae	x		
Maíz chaucha	Maize	*Zea mays* L.	Poaceae	x		
Maíz chulpe	Maize	*Zea mays* L.	Poaceae	x	x	x
Maíz diente de caballo	Maize	*Zea mays* L.	Poaceae			x
Maiz guandango	Maize	*Zea mays* L.	Poaceae	x		x
Maíz guandango blanco	Maize	*Zea mays* L.	Poaceae	x		
Maíz mishka	Maize	*Zea mays* L.	Poaceae	x		x
Maíz morocho blanco	Maize	*Zea mays* L.	Poaceae			x
Maíz morocho uructuco de colores	Maize	*Zea mays* L.	Poaceae	x		
Maíz negro	Maize	*Zea mays* L.	Poaceae			x
Mandarina	Mandarin	*Citrus reticulata* Blanco	Rutaceae	x	x	
Manzana	Apple	*Malus pumila* Mill.	Rosaceae	x		
Manzanilla de Castilla	Chamomile	*Matricaria chamomilla* L.	Asteraceae		x	
Maracuyá morada	Passion fruit	*Passiflora edulis* Sims	Passifloraceae	x		
Mashua		*Tropaeolum tuberosum* Ruiz & Pav.	Tropaeolaceae	x	x	x
Mashua negra		*Tropaeolum tuberosum* Ruiz & Pav.	Tropaeolaceae	x		
Melloco	Melloco	*Ullucus tuberosus* Caldas	Basellaceae	x	x	x
Melloco blanco	Melloco	*Ullucus tuberosus* Caldas	Basellaceae	x		
Menta	Mint	*Mentha* x *piperita* L.	Lamiaceae		x	x
Miso		*Mirabilis expansa* (Ruiz & Pav.) Standl.	Nyctaginaceae			x
Mora de Castilla	Blackberry	*Rubus glaucus* Benth.	Rosaceae	x		x
Mora sin espina	Blackberry	*Rubus* sp.	Rosaceae	x		
Motilón		*Hieronyma macrocarpa* Müll.Arg.	Phyllanthaceae	x		
Muku chaklla		*Piper hispidum* Sw.	Piperaceae		x	
Nabo	Turnip	*Brassica napus* L.	Brassicaceae		x	
Nabo chino	Chinese turnip	*Brassica rapa* L.	Brassicaceae	x		
Naranja	Orange	*Citrus sinensis* (L.) Osbeck	Rutaceae	x	x	
Naranjilla		*Solanum quitoense* Lam.	Solanaceae	x	x	
Niguas-pikiyuyo		*Margyricarpus pinnatus* (Lam.) Kuntze	Rosaceae		x	
Ocas	Oca	*Oxalis tuberosa* Molina	Oxalidaceae	x	x	x
Orégano macho	Oregano	*Origanum* sp.	Lamiaceae			x
Ortiga negra	Stinging nettle	*Urtica dioica* L.	Urticaceae		x	
Paico		*Chenopodium ambrosioides* L.	Amaranthaceae		x	
Palma de ramos	Wax palm	*Ceroxylon echinulatum* Galeano	Arecaceae		x	
Papa Anita	Potato	*Solanum tuberosum* L.	Solanaceae			
Papa arag	Potato	*Solanum tuberosum* L.	Solanaceae			x
Papa cachito	Potato	*Solanum tuberosum* L.	Solanaceae	x		
Papa calavera	Potato	*Solanum tuberosum* L.	Solanaceae	x		
Papa Canadá	Potato	*Solanum tuberosum* L.	Solanaceae	x		
Papa chaucha	Potato	*Solanum tuberosum* L.	Solanaceae			x
Papa chaucha negra	Potato	*Solanum tuberosum* L.	Solanaceae		x	x
Papa chaucha ojos blancos	Potato	*Solanum tuberosum* L.	Solanaceae			x
Papa china	Potato	*Solanum tuberosum* L.	Solanaceae		x	
Papa chola	Potato	*Solanum tuberosum* L.	Solanaceae			x
Papa curipamba	Potato	*Solanum tuberosum* L.	Solanaceae			x
Papa holandesa	Potato	*Solanum tuberosum* L.	Solanaceae	x		x
Papa leona blanca	Potato	*Solanum tuberosum* L.	Solanaceae		x	
Papa moronga	Potato	*Solanum tuberosum* L.	Solanaceae		x	
Papa pan de azúcar	Potato	*Solanum tuberosum* L.	Solanaceae		x	
Papa parda	Potato	*Solanum tuberosum* L.	Solanaceae		x	
Papanabo		*Brassica napus* L.	Brassicaceae	x	x	
Papaya	Papaya	*Carica papaya* L.	Caricaceae		x	
Pataconyuyo		*Peperomia* sp.	Piperaceae		x	
Penco de Castilla		*Furcraea andina* Trel.	Asparagaceae		x	
Penco azul	Agave	*Agave americana* L.	Asparagaceae		x	
Pera	Pear	*Pyrus communis* L.	Rosaceae	x		
Perejil	Parsley	*Petroselinum crispum* (Mill.) Fuss	Apiaceae		x	
Pimiento	Bell peppers	*Capsicum annuum* L.	Solanaceae		x	
Plátano dominico	Plantain	*Musa* x *paradisiaca* L.	Musaceae	x		
Porotón		*Erythrina edulis* Micheli	Fabaceae	x		
Pumamaqui		*Oreopanax ecuadoriensis* Seem.	Araliaceae		x	
Pumarrosa		*Syzygium jambos* (L.) Alston	Myrtaceae	x		
Quinua	Quinoa	*Chenopodium quinoa* Willd.	Amaranthaceae	x	x	
Quinua dulce	Quinoa	*Chenopodium quinoa* Willd.	Amaranthaceae		x	
Rábano	Radish	*Raphanus sativus* L.	Brassicaceae		x	
Rábano silvestre	Wild radish	*Raphanus sativus* L.	Brassicaceae		x	
Rocoto	Chili	*Capsicum pubescens* Ruiz & Pav.	Solanaceae		x	
Romero	Rosemary	*Rosmarinus officinalis* L.	Lamiaceae		x	x
Rondobalín		*Salpichroa diffusa* Miers	Solanaceae		x	
Ruda	Rute	*Ruta graveolens* L.	Rutaceae		x	
Sábila	Aloe	*Aloe vera* (L.) Burm.f.	Xanthorrhoeaceae	x		
Sambo	Squash	*Cucurbita* sp.	Cucurbitaceae	x		
Sambo tsímbalo		*Cucurbita* sp.	Cucurbitaceae			x
Santa María		*Tanacetum parthenium* (L.) Sch.Bip.	Asteraceae		x	
Sauco		*Sambucus nigra* L.	Adoxaceae		x	
Símbalo		*Solanum* sp.	Solanaceae		x	x
Sunfo		*Satureja nubigena* (Kunth) Briq.	Lamiaceae		x	
Tarugacacho		*Halenia weddelliana* Gilg	Gentianaceae		x	
Taxo	Banana passion fruit	*Passiflora tripartita* (Juss.) Poir	Passifloraceae		x	x
Tigricillo		*Iresine herbstii* Hook.	Amaranthaceae		x	
Tilo		*Sambucus peruviana* Kunth	Adoxaceae		x	
Tipo blanco		*Minthostachys mollis* (Benth.) Griseb.	Lamiaceae		x	
Tomate de árbol	Purple tree tomato	*Solanum betaceum* Cav.	Solanaceae	x	x	
Tomate de árbol morado	Tree tomato	*Solanum betaceum* Cav.	Solanaceae			x
Tomate riñón	Tomato	*Solanum lycopersicum* L.	Solanaceae	x	x	x
Tomillo	Thyme	*Thymus vulgaris* L.	Lamiaceae		x	
Toronjil	Melissa	*Melissa officinalis* L.	Lamiaceae			x
Trigo	Wheat	*Triticum vulgare* Vill.	Poaceae	x	x	x
Trigo centeno	Wheat	Not identified	Poaceae	x	x	x
Trigo crespo	Wheat	Not identified	Poaceae	x		
Trigo isobamba	Wheat	*Triticum vulgare* Vill.	Poaceae	x		
Trueno		Not identified			x	
Uva	Grape	*Vitis vinífera* L.	Vitaceae	x	x	
Uvilla	Golden berry	*Physalis peruviana* L.	Solanaceae	x		x
Valleco (herb)		Not identified		x		
Verbena hembra		*Verbena officinalis* L.	Verbenaceae		x	
Vervena macho		*Verbena officinalis* L.	Verbenaceae		x	
Wagra habas		*Vicia faba* L.	Fabaceae			x
Wagra ortiga		*Urtica* sp.	Urticaceae		x	
Yanacara		*Tournefortia fuliginosa* Kunth	Boraginaceae		x	
Zanahoria amarilla	Arracacha	*Arracacia xanthorrhiza*	Apiaceae	x		x
Zanahoria blanca	Arracacha	*Arracacia xanthorrhiza* Bancr.	Apiaceae	x		x
Zanahoria limeña	Arracacha	*Arracacia* sp.	Apiaceae	x		
Zanahoria morada	Arracacha	*Arracacia* sp.	Apiaceae	x	x	
Zapallo	Pumpkin	*Cucurbita* sp.	Cucurbitaceae	x		
Zapallo grande amarillo	Pumpkin	*Cucurbita* sp.	Cucurbitaceae	x		
Zapallo kiliko	Pumpkin	*Cucurbita* sp.	Cucurbitaceae	x		
Zapallo verde de masa	Pumpkin	*Cucurbita* sp.	Cucurbitaceae	x		
Zorrojigua		Not identified			x	
